# The Effects of Depth of Anesthesia on Muscle-Recorded Motor Evoked Potentials: A Prospective Observational Study

**DOI:** 10.1213/ANE.0000000000007777

**Published:** 2025-11-20

**Authors:** Maria C. Gadella, Marko M. Sahinovic, Sebastiaan E. Dulfer, Katalin Tamási, Fiete Lange, Cristopher Faber, Frits Hein Wapstra, Rob J. M. Groen, Anthony R. Absalom, Gea Drost

**Affiliations:** From the Departments of 1 Anesthesiology; 2 Neurosurgery; 3 Epidemiology; 4 Neurology; 5 Orthopedics, University Medical Center Groningen, University of Groningen, Groningen, the Netherlands; 6 Department of Neurosurgery, Faculty of Medicine Universitas Airlangga, Dr. Soetomo General Academic Hospital, Surabaya, Indonesia.

## Abstract

**BACKGROUND:**

Intraoperative neurophysiological monitoring is used to assess neurological function during surgeries placing the spinal cord at risk. Transcranial electrical stimulation muscle motor evoked potentials (Tc-mMEPs) are used to monitor motors tracts, but their interpretation is complicated by the large temporal variability which can result in false-positive warnings. Although the choice of anesthetic drug regimen and drug dose are often claimed to be factors causing this variability, the relationship between depth of anesthesia, quantified by processed electroencephalogram (pEEG) parameters, and Tc-mMEPs characteristics in upper and lower extremity muscles have not yet been rigorously investigated in patients receiving propofol/remifentanil-based anesthesia.

**METHODS:**

Twenty-five patients were included in this prospective observational study. All received propofol/remifentanil-based total intravenous anesthesia. Depth of anesthesia was quantified by the bispectral index (BIS). After induction of anesthesia, the target propofol concentration was altered to sequentially achieve BIS values of either 30, 40, and 50, or the reverse (direction randomly determined). At each depth of anesthesia Tc-mMEP thresholds were determined, and arterial blood samples were collected. Supramaximal Tc-mMEP signals were recorded every 2 minutes and amplitudes, latencies and area under the curve (AUC) were subsequently calculated. Effects of depth of anesthesia on Tc-mMEP outcomes were analyzed using linear mixed effects modeling.

**RESULTS:**

The median (range) age of the study population was 18 (14–66) years (n = 25). In the leg muscles, a decrease of 10 BIS points was associated with a decrease in Tc-mMEP amplitude of 11%–12% (all *P* < .001; mean [95% confidence interval {CI}], 12% [7.1–16], 11% [6.8–16], and 12% [7.5–16], for the AH, TA, and GAS muscles, respectively). In contrast, no significant amplitude or AUC change was found in the hand muscles (*P* = .201, 2.8% [−1.5 to 7.1] and *P* = .076, 4.0% [−0.4 to 7.6], respectively). Latencies changed <0.5% per 10 BIS points decrease (0.03% [−0.3 to 0.2], −0.2% [−0.5 to 0.1], −0.2% [−0.5 to 0.04], 0.3% [0.04–0.6] for the AH, TA, GAS, and hand muscles, respectively), and thresholds increased 3.6% (0.8–7) when BIS decreased from 50 to 30 (*P* = .037).

**CONCLUSIONS:**

Our findings challenge some commonly held beliefs. First, our findings suggest that deeper anesthesia has differential effects on the different muscle groups, with little effect on the hand muscles. The current practice of using the hand signals as reference values during procedures below C8/T1 may therefore need re-evaluation. Second, the paucity of effect of depth of anesthesia on Tc-mMEP thresholds and latencies suggests that Tc-mMEP generation is not influenced by deep anesthesia in a clinically relevant way. Therefore, the threshold level monitoring method may provide a more reliable indicator of motor pathway integrity during surgery. This could reduce the likelihood of false-positive warnings and unnecessary interventions.

**KEY POINTS**

- **Question:** How does the depth of anesthesia, quantified by bispectral index (BIS) values, affect the characteristics of transcranial electrical stimulation muscle motor evoked potentials (Tc-mMEPs) in patients receiving propofol/remifentanil-based anesthesia?
- **Findings:** There is a significant association between BIS values and Tc-mMEP amplitudes and area under the curve (AUC) values in the leg muscles, but not in the hand muscles.
- **Meaning:** Deeper anesthesia has differential effects on different muscle groups, with little effect on the hand muscles, suggesting that hand signals may not be reliable reference values during procedures below C8/T1, and the minimal influence of anesthesia depth on Tc-mMEP thresholds and latencies suggests that threshold level monitoring may provide a more reliable indicator of motor pathway integrity during surgery, potentially reducing the likelihood of false-positive warnings and unnecessary interventions.

**See Article, page 730**

Intraoperative neurophysiological monitoring (IONM) is used to assess the functional integrity of neurological structures.^1,2^ When transcranial electrical stimulation (Tc) is applied, motor tract potentials are recorded over peripheral muscles (muscle motor evoked potentials [mMEPs]). Tc-mMEP amplitudes exhibit high within-person variability, which can result in false warnings and unnecessarily delay in surgical procedure. While false-negative Tc-mMEP results are rare, reported incidence of false-positive warning rates is 1.9%–14.7%.^3–5^

The anesthetic drug regimen and drug doses are often reported to be key factors influencing Tc-mMEP reliability.^2,6–9^ Multiple studies have shown that muscle relaxants and inhalational anesthetics depress Tc-mMEP amplitudes.^7,9–12^ Total intravenous anesthesia (TIVA) with propofol and opioid infusions also affects Tc-mMEPs amplitudes, but to a lesser extent than volatile regimens.^13^ Previous research showed that propofol boluses can cause a decrease in Tc-mMEP amplitude^14^ and that increasing propofol infusion rates and plasma concentrations are associated with decreasing Tc-mMEP amplitudes, while latencies remain unchanged.^15–17^ Propofol infusion rates and plasma concentrations are however weak surrogates of anesthetic depth. First, there is a high variability in the pharmacokinetic relationships between drug doses and plasma concentration. Second, there is very broad variability in the pharmacodynamic relationship between plasma propofol concentration and the actual clinical effect.^18^ For this reason, processed electroencephalogram (pEEG) depth of anesthesia monitors, such as the bispectral index (BIS), are often used in clinical practice to provide an objective measure of clinical effect and help guide anesthetic dose.^18,19^ To our knowledge, only one study has evaluated the effect of depth of anesthesia quantified using a pEEG parameter, on Tc-mMEP characteristics, but this study involved only one muscle, and propofol-only anesthesia (which is uncommon in clinical practice).^20^

The primary objective of this study was therefore to investigate the relationship between depth of propofol/remifentanil anesthesia, quantified by the BIS, and the characteristics of Tc-mMEP signals from upper and lower limb muscles. The secondary objectives were (A) to compare models using BIS and propofol variables as predictors for Tc-mMEP amplitude and (B) to assess if similar conclusions were reached if these models were corrected for blood pressure and propofol plasma concentration.

## METHODS

This study was conducted in accordance with the Declaration of Helsinki and was reviewed and approved by the Ethics Committee of the University Medical Center Groningen (METc 2018/630). The study protocol followed the terms of the Dutch Act on Medical Research on Human Subjects (Wet Medisch Wetenschappelijk Onderzoek, or “WMO”). All patients gave informed consent for participation in this prospective observational study. The trial was registered before enrollment at the Dutch trial registry (NL-OMON48128, Principal investigator: R.J.M.G., date of registration: March 1, 2019). The study protocol was published before the start of inclusion.^21^

### Patients

Patients of age 12 years and older who were planned for spinal surgery with Tc-mMEP spinal cord monitoring were eligible for the study. Exclusion criteria were (1) preoperative weakness in one or more of the following lower extremity muscles; tibialis anterior (TA), gastrocnemius (GAS), or abductor hallucis (AH), (2) a medical history of epilepsy, severe cardiac disorders, or coronary/carotid artery disease, or (3) implantation of pacemaker, implantable cardioverter-defibrillator (ICD), or cerebral metals.

### Study Objectives

The objective of this study was to prospectively investigate the relationship between depth of anesthesia, quantified as the BIS, and the characteristics of Tc-mMEP signals. The secondary objectives were divided into 2 parts. In pEEG Versus Propofol Concentrations Models the objective was to compare models using BIS versus propofol concentrations—calculated or measured—as predictors for Tc-mMEP amplitude. In pEEG Only versus More Complex Models, the objective was to evaluate if similar conclusions were reached if the models were corrected for blood pressure and propofol calculated plasma concentration. All study measurements were performed before incision to minimize nociceptive stimuli and to clearly distinguish between the effects of changes in depth of anesthesia and surgically induced Tc-mMEP changes.

### Anesthetics Management

Anesthetic management was in accordance with study and departmental protocols. Anesthesia was achieved and maintained by target-controlled infusions of propofol (Schnider model^22^) and remifentanil (Minto model^23^). Inhalational anesthetic agents were not used, except for the case of 1 patient who briefly received low doses of sevoflurane while venous access was achieved before induction of anesthesia with propofol and remifentanil. Muscle relaxants were only given during the induction of anesthesia to facilitate endotracheal intubation, but not thereafter to avoid negative effects on Tc-mMEPs. Before the first study measurements, train-of-four (TOF) testing was performed in all patients to determine the degree of neuromuscular block. If the strength of the fourth twitch was >80% of that of the first twitch, the effects of neuromuscular blockade were considered negligible. Sugammadex was given to 4 patients to reach the desired TOF score before study measurements. Electrodes required for generation and recording of the Tc-mMEPs were applied after induction of anesthesia.^24^ The study-related interventions and measurements began after prone positioning on the operating table. Before surgery, the random assignment function in SPSS (IBM SPSS statistics version 28.0.0.0) was used to randomly assign each patient to 1 of 2 sequences: BIS 50–40–30 or BIS 30–40–50. For each phase, the anesthesiologist manipulated the target propofol effect-site concentration to achieve the desired BIS value. As the BIS fluctuates considerably during anesthesia, we considered the depth of anesthesia to be acceptably on target if the BIS value was consistently within 5 points of the desired value for at least 2 minutes, following which the study measurements were performed. Three blood samples for subsequent plasma propofol concentration assays were taken at each BIS level (30, 40, and 50). No ketamine was given during the study period. During the study measurements, we attempted to maintain the mean arterial blood pressure and core body temperature as stable as possible and close to 70 mm Hg and 37 °C, respectively. After the study was completed, the propofol administration and other aspects of anesthetic management were at the discretion of the attending anesthesiologist.

### Transcranial Electrical Stimulation Muscle Motor Evoked Potentials

Transcranial electrical stimuli were applied at locations Cpl1–Cpl2 (1 cm posterior and 1 cm lateral to C1 and C2) using a constant voltage stimulator (NIM-Eclipse E4 IONM system; Medtronic BV). Stimulation parameters were set as follows: pulse duration was 75 μs, single train stimulation was applied, number of pulses per train were 5, and inter-stimulus interval that produced highest amplitudes was determined per patient. Tc-mMEP thresholds were assessed using 10 V increments and were defined as the voltage required to elicit an amplitude detectable at a 50 μV window setting. Thresholds were measured 3 times at steady state BIS levels of 30, 40, and 50. The Tc-mMEP amplitudes were obtained using supramaximal stimulation and were recorded every 2 minutes. Supramaximal was defined as the stimulation intensity for which no more increase in amplitude occurred in all muscles. Tc-mMEPs for the lower extremities were recorded using surface electrodes placed on the TA, GAS, and AH muscles on both the left and right sides. For the upper extremities, surface electrodes were placed bilaterally on the abductor pollicis brevis (APB) and abductor digiti minimi (ADM) muscles, producing a combined hand muscle (HAND) response.

### Data Collection

Tc-mMEP signals were exported from the NIM-Eclipse E4 IONM system (Medtronic BV), whereafter the Tc-mMEP amplitudes and area under the curves (AUCs) were calculated and recorded using Python (version 3.7.1) software routines. Tc-mMEP latencies were determined using a peak analysis script written in MatLab R2023a. All latencies were manually checked and adjusted when necessary. Anesthetic data were automatically downloaded electronically every 15 seconds. Anesthetic variables of interest were subsequently extracted from the hospital information system. Mean arterial pressure (MAP) and BIS values were smoothed using a moving median filter of 265 seconds to filter out artifacts (ie, flushing after taking blood samples). Curves of each anesthetic and IONM parameter were visually inspected for artifacts. The consecutive Tc-mMEP amplitudes, AUCs, latencies, and thresholds were adjusted to the values corresponding to the measurement nearest to a BIS value of 40 for each patient and muscle (Formula 1). This adjustment was performed to account for absolute differences between randomization groups. All continuous outcome and predictor variables were centered to make the models more robust against multicollinearity.


$$\begin{array}{l} \frac{Tc-mMEP\;outcome\;value\;at\;BIS=\left[x\right]}{Tc-mMEP\;outcome\;value\;at\;BIS=40}. \end{array}$$ (1)

### Statistical Analysis

Since there were insufficient data available on which to base a sample size calculation, a pragmatic sample size of 25 patients was chosen. A post-hoc power calculation is however presented in Supplemental Digital Content 1, Supplement A, https://links.lww.com/AA/F492. All analyses were conducted using R Software version 4.2.2 (The R Foundation for Statistical Computing). Log transformation was applied to non-normally distributed variables, where it improved model fit. Linear mixed effects models were used to analyze the repeated Tc-mMEP measurements, using the “lmer” function from the lme4 package in R.

For the primary objective, the association between depth of anesthesia, quantified as the BIS, and Tc-mMEP outcomes—amplitude, AUC, and latency—was examined by including BIS as a continuous variable. In the threshold model, BIS was treated as a categorical variable due to data collection at 3 predefined BIS levels (30, 40, and 50). To correct for repeated measurements, patient level was included as a random intercept in all models. Muscle, side, and randomization group were incorporated as fixed effects. BIS was added as a random slope where the model converged and exhibited significant improvement. Significant model improvement was defined as an analysis of variance (ANOVA) test outcome of *P* < .05. Interactions between BIS, muscle, and side were included if they significantly enhanced model fit.

**Table 1.** Patient Characteristics and Pharmacological and Physiological Parameters

Characteristics	Patients (n = 25)			
Age (median [range])	18 [14–66]			
Female, N (%)	15 (60)			
Height (cm) (mean ± SD)	172 ± 10			
Weight (kg) (mean ± SD)	63.8 ± 17.7			
BMI (mean ± SD)	21.3 ± 4.4			
Randomization group, N				
Increasing depth of anesthesia	13			
Decreasing depth of anesthesia Diagnosis, N	12			
Orthopedic surgery				
Idiopathic scoliosis/kyphosis	20			
Neurosurgery				
Spinal extradural tumor	2			
Spinal intradural extramedullary tumor	2			
Spinal intradural intramedullary tumor	1			
Pharmacological and physiological parameters	BIS 30	BIS 40	BIS 50	% Missing
Propofol Ce (µg/mL) (mean ± SD)	3.3 ± 0.8	2.6 ± 0.6	2.0 ± 0.4	21.51
Propofol Cp (mean ± SD)	3.2 ± 1.1	2.6 ± 0.9	2.2 ± 0.8	19.59
Propofol Ct (mean ± SD)	3.1 ± 0.8	2.5 ± 0.7	2.0 ± 0.5	21.51
Remifentanil Ce (ng/mL) (median [IQR])	4.0 [4.0–4.0]	4.0 [4.0–4.0]	4.0 [4.0–4.0]	21.51
MAP mm Hg (mean ± SD)	68.0 ± 7.0	68.4 ± 7.6	68.3 ± 7.2	0.64
Norepinephrine (µg/kg/min) (median [IQR])	0.018 [0.012–0.026]	0.018 [0.010–0.027]	0.013 [0.010–0.026]	22.47
BIS (mean ± SD)	29 ± 4	40 ± 3	52 ± 5	0.00
Spo_2_ (%) (median [IQR])	98 [98–99]	98 [97–99]	98 [97–99]	0.00
Temperature (°C) (mean ± SD)	36.2 ± 0.4	36.2 ± 0.4	36.2 ± 0.4	4.47
Heartrate (bpm) (mean ± SD)	56.4 ± 7.3	56.3 ± 7.9	56.4 ± 7.4	0.00
Cardiac output (L/min) (mean ± SD)	3.5 ± 1.2	3.5 ± 1.2	3.2 ± 1.0	5.67
Stroke volume variation (%) (mean ± SD)	12.1 ± 3.0	12.3 ± 3.5	13.3 ± 3.1	2.47
End tidal CO_2_ (kPa) (mean ± SD)	4.4 ± 0.3	4.4 ± 0.3	4.4 ± 0.3	0.00

Abbreviations: BIS, bispectral index; BMI, body mass index; Ce, calculated effect-site concentration; Cp, calculated plasma concentration; Ct, target effect-site concentration; IQR, interquartile range; MAP, mean arterial pressure; SD, standard deviation; Spo_2_, peripheral oxygen saturation.

**Table 2.** Amplitude Changes and Warning Criteria per Muscle

	BIS 50–BIS 30		
Muscle	Min.^a^	Max.^a^	Warning criteria^b^ (n)
GAS	−0.319	4.054	2
AH	−0.276	2.736	5
TA	−0.592	1.126	2
HAND	−0.498	3.592	4

Abbreviations: AH, abductor halluces; BIS, bispectral index; GAS, gastrocnemius; HAND, hand muscle; TA, tibialis anterior.

a Values are proportions of the transcranial electrical stimulation muscle motor evoked potential amplitude when BIS was closest to 40.

b Number of patients in which warning criteria were met. Criterium of >50% amplitude decrease.

**Table 3.** Results of Primary Objective

Outcome	Percentage change per 10 BIS points		95% CI	*P* value
Amplitude^abc^				
AH	12.03		7.05–16.44	**<.001**
TA	11.21		6.82–15.62	**<.001**
GAS	11.89		7.49–16.29	**<.001**
HAND	2.81		−1.50 to 7.14	.201
AUC^ab,c^				
AH	12.07		8.03–16.13	**<.001**
TA	11.06		7.03–15.11	**<.001**
GAS	13.09		9.04–17.14	**<.001**
HAND	4.01		−0.38 to 7.62	.076
Latency^ac,d^				
AH	−0.03		−0.31 to 0.22	.749
TA	−0.20		−0.47 to 0.07	.145
GAS	−0.24		−0.53 to 0.04	.092
HAND	0.31		0.04–0.58	**.022**
Outcome	**Percentage change compared to reference category**		95% CI	*P* value
Threshold^a,e^ (V)	BIS 30	Reference category		
	BIS 40	−1.193	−3.086 to 5.968	.197
	BIS 50	−3.562	−7.002 to −0.813	**.037**

Bold values indicate statistically significant values.

Abbreviations: AH, abductor hallucis muscle; AUC, area under the curve; BIS, bispectral index; CI, confidence interval; GAS, gastrocnemius muscle; HAND, hand muscle; TA, tibialis anterior muscle.

a Proportion of BIS = 40 transcranial electrical stimulation muscle motor evoked potential outcomes, corrected for side, muscle, and randomization group.

b Continuous log-transformed outcome data were added to the model.

c Interaction between muscle and BIS was added.

d Interaction between muscle and side was added.

e BIS was added as a random slope.

**Figure 1.**
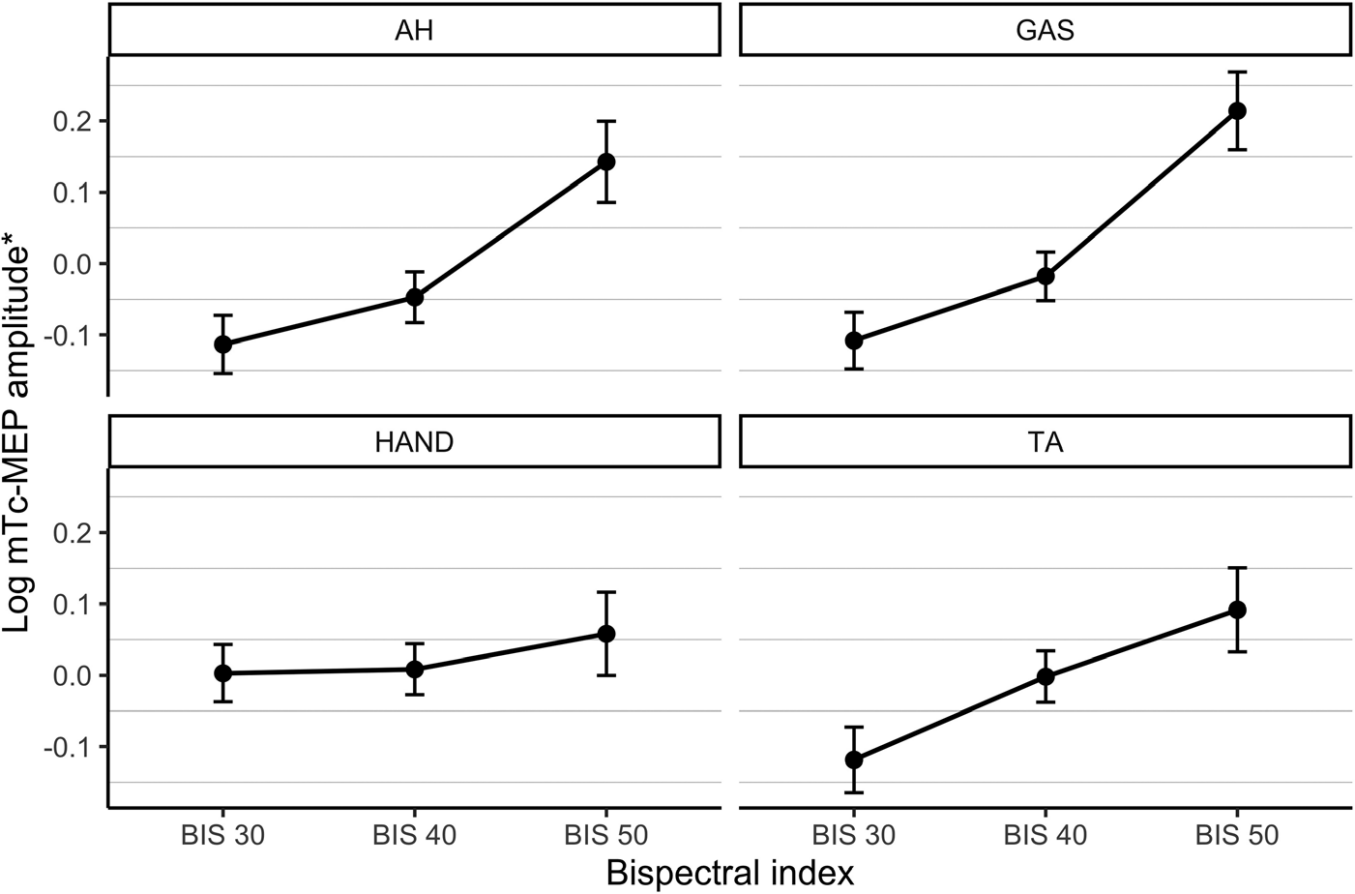
Relationship between BIS values and log-transformed Tc-mMEP amplitudes and Tc-mMEP thresholds per muscle. *Proportion of the values corresponding to the measurement nearest to a BIS value of 40 per patient and muscle. AH indicates abductor hallucis muscle; BIS, bispectral index; GAS, gastrocnemius muscle; HAND, hand muscle; TA, tibialis anterior muscle; Tc-mMEP, transcranial electrical stimulation muscle motor evoked potential.

**Table 4.** BIS Versus Propofol Concentrations Models

Variable	AIC	SD	Delta AIC	Conditional *R*^2^
BIS	3723.680	0	0.000	0.400
Propofol target concentration	3726.970	5.372	3.290	0.377
Propofol calculated effect-site concentration	3727.921	4.779	4.241	0.379
Propofol calculated plasma concentration	3752.004	5.784	28.324	0.375
Propofol measured plasma concentration	3749.140	7.899	25.460	0.386

Abbreviations: AIC, Akaike information criterion; BIS, bispectral index; SD, standard deviation.

To address missing data for the secondary objectives, multiple imputation was performed using the “mice” package^25^ with predictive mean matching (pmm). PMM was used because multilevel imputation was not feasible, as some subjects had no (n = 1) or very few (n = 4) values for drug infusion rates. A total of 20 imputed datasets were analyzed and pooled using Rubin’s rules to account for imputation variability. For the secondary objectives, the model structure from the primary objective was retained, and adjustments were made solely to the predictors. In pEEG Versus Propofol Concentrations Models, model performance was evaluated using the Akaike Information Criterion (AIC) and conditional *R*^2^ to assess improvements in fit when BIS was replaced as predictor from the original model by propofol target-, effect site-, calculated plasma-, or measured plasma concentrations. An absolute difference in AIC (delta AIC) of <2 was considered not relevant while values between 2 and 10 were considered possibly relevant and >10 were considered relevant (considerable differences in explained variability between the models).^26^ Lowest AIC value indicates the “best” model. For models without imputation, conditional *R*² values were estimated using the *r.squaredGLMM*() function from the MuMIn package.^27^ For models based on multiple imputed datasets, *R*² values were computed separately per imputation using the same function and subsequently pooled using Fisher’s Z-transformation and Rubin’s rules.^28^ In pEEG Only versus More Complex Models, model improvement was assessed by incorporating MAP and propofol calculated plasma concentration one by one to the primary model. When model fit significantly improved, the new variable was kept in the model and the next variable was added to check for further model improvement.

## RESULTS

### Patients

Twenty-five consecutive patients were included between November 2022 and March 2024. Optimal interstimulus intervals (ISI) were 1.5 ms for 19 patients, 1 ms for 5 patients, and 3 ms for 1 patient. Patient characteristics, pharmacological and physiological parameters are summarized in Table 1.

### Missing Data

In Table 1, missing data percentages for pharmacological and physiological parameters are described. The reasons for missing data are as follows. A proportion of data for the propofol, remifentanil, and noradrenaline infusion rates is missing, because of intermittent connectivity problems in the Wi-Fi system transmitting these variables to the electronic patient record . Propofol concentration assays were not possible for 5 patients (20%). In these cases, blood was acquired and the samples appropriately labelled and stored, but an organizational failure led to their samples being discarded. Finally, data from muscles that exhibited absent or inconsistent Tc-mMEP responses were excluded from the analysis. Specifically, one patient with an extradural tumor exhibited no responses in both the GAS muscles and left AH and TA muscles, while the right TA muscle responses were variably elicitable. Another patient who underwent scoliosis surgery had variable responses in the right-hand and right AH muscles. All other patients demonstrated reliable responses in all muscles and a total of 2506 supramaximal Tc-mMEP responses and 576 thresholds were included for the primary objective analysis.

### Primary Objective

Figure 1 displays the relationship between BIS and log transformed Tc-mMEP amplitudes and Tc-mMEP thresholds for 4 different muscles. The range of amplitude change between BIS 50 and BIS 30 is shown in Table 2. The data suggest that decreasing the BIS from 50 to 30 would have resulted in false-positive warnings (50% amplitude reduction criterium) in 5 patients for the AH muscle, 4 patients for the HAND, and 2 patients for the GAS and TA muscles. A case example of a patient with clearly visible amplitude decreases when BIS gradually changed from 50 to 30 is presented in Supplemental Digital Content 2, Supplement B, https://links.lww.com/AA/F493. Linear mixed effects model analysis showed significant associations between BIS and Tc-mMEP amplitudes and AUC in the leg muscles (all *P* < .001) (Table 3). A decrease of 10 BIS points was associated with 11%–13% decrease in Tc-mMEP amplitudes and AUC of the lower extremity muscles. The Tc-mMEP amplitude decreased 12% (95% confidence interval [CI], 7.1–16), 11% (95% CI, 6.8–16), and 12% (95% CI, 7.5–16) per 10 BIS points decrease for the AH, TA, and GAS muscles, respectively. The AUC decreased 12% (95% CI, 8.0–16), 11% (95% CI, 7–15), and 13% (95% CI, 9.0–17) per 10 BIS points decrease for the AH, TA, and GAS muscles, respectively. No significant association between BIS and Tc-mMEP amplitudes or AUC in the HAND was found (95% CI, −1.5 to 0.7, *P* = .201 and 95% CI, −0.4 to 8, *P* = .076, respectively). Moreover, no significant association between latency and BIS was found for the leg muscles (95% CI, −0.3 to 0.2, −0.5 to 0.1, and −0.5 to 0.04 for the AH, TA, and GAS muscles, respectively). The HAND had a 0.31% shorter latency with a decrease of 10 BIS points (95% CI, 0.04–0.6; *P* = .022). Voltage thresholds were not significantly lower when BIS was 40 compared to BIS values of 30 (95% CI, −3.1 to 6.0; *P* = .197). When BIS was 50, thresholds were 3.6% lower than when BIS was 30 (95% CI, −7.0 to −0.8; *P* = .037) (Table 3).

### Secondary Objectives

#### pEEG Versus Propofol Concentrations Models

Table 4 shows the changes in AIC and conditional *R*^2^ when BIS, initially used as a predictor in the primary amplitude model, is replaced by various propofol variables (estimated or measured). If multiple imputation was used, the mean AIC is presented. The AIC values for each predictor reflect the overall model fit, and lower values indicate better model performance. Higher *R*^2^ values indicate better model fit. The primary model with BIS as predictor had the lowest AIC value and highest conditional *R*^2^ value, and can therefore be considered the best model to use to predict Tc-mMEP amplitude. Models with propofol target concentration or estimated effect-site concentration as predictors had AIC values that were 3.29–4.24 points higher, compared to the model using BIS as a predictor. Models with estimated or measured plasma propofol concentration as predictor had AIC values that were 28 and 25 points higher than the model with BIS as predictor. The conditional *R*^2^ values were lowest for the model with propofol calculated plasma concentration as predictor and had an absolute difference of 0.025 compared to the model with BIS as predictor. Complete case model results are available in the Supplemental Digital Content 3, Supplement C, https://links.lww.com/AA/F494.

#### pEEG Only versus More Complex Models

Adding propofol calculated plasma concentration and MAP to the basic model significantly improved the model performance for amplitude and AUC linear mixed effects models. Lower extremity Tc-mMEP amplitudes and AUCs increased 7%–12% per 10 BIS points when corrected for these variables (all *P* < .001, 95% CI, 5.3–14 and 6.2–14 for the AH muscles, 2.8–12 and 3.3–12 for the TA muscles, and 7.6–17 and 7.8–16 for the GAS muscles, respectively; Table 5). HAND amplitudes and AUC increased 3% per 10 BIS points and were not significantly associated with BIS (*P* = .200, 95% CI, −1.5 to 7.3 and *P* = .119, 95% CI, −0.8 to 7.3, respectively). The models with latency or thresholds as outcome variable did not show significant improvement when MAP or propofol calculated plasma concentrations were added as variables. Complete case model results are available in the Supplemental Digital Content 3, Supplement C, https://links.lww.com/AA/F494.

## DISCUSSION

In this study, we examined the effects of depth of propofol/remifentanil anesthesia, quantified by pEEG, on Tc-mMEPs. Deep anesthesia significantly decreased Tc-mMEP amplitudes and AUCs in leg muscles but not in the HANDs. Specifically, a decrease in 10 BIS points corresponded with a 11%–13% decline of leg Tc-mMEP amplitudes and AUCs (all *P* < .001), whereas hand Tc-mMEP signals decreased by 3%–4% (*P* = .201 and .076, respectively). Notably, no clinically relevant change in thresholds or latencies occurred when depth of anesthesia was altered. At BIS 50, thresholds were 3.6% lower compared to BIS 30, and latencies changed <0.5% per 10 BIS units.

Previous research showed inverse associations between propofol infusion rates or plasma concentrations and Tc-mMEP amplitude^14–17,29^ but no significant association with latencies.^15^ Our study is the first examining the effects of depth of intravenous anesthesia on Tc-mMEP thresholds. Only one previous study investigated the influence of depth of anesthesia and showed stable Tc-mMEP amplitudes within a BIS range of 25–65, but no opioids were used, and only a limited number of measurements were made, and then only from the right TA muscle, using an unclear application of supramaximal stimulation.^20^

The variability in Tc-mMEP amplitudes and false warning incidences is influenced by the monitoring technique and the warning criteria used. Body temperature, blood pressure, and blood oxygenation can also affect Tc-mMEP signals.^2,30–35^ It is essential to consider these additional factors to accurately interpret IONM results. Our secondary objective results show that BIS values correlate with Tc-mMEP amplitudes. Propofol effect-site and target concentration can also serve as predictors for amplitude. The associations identified between depth of anesthesia and Tc-mMEP characteristics remained largely consistent in more complex models incorporating blood pressure and propofol concentration as covariates, suggesting minimal to no confounding effects of these variables on the results.

When the amplitude reduction Tc-mMEP monitoring method is used, an 50%–100% amplitude decrease is generally considered a warning, whereas when the threshold level method is used, an increase of >100 V is typically classified as a warning.^2,36^ Our results suggest that deep anesthesia can cause significant leg muscle amplitude decreases, potentially leading to false-positive warnings with the amplitude reduction method. In contrast, false-positive warnings due to deep anesthesia seem unlikely with the threshold level method.

During IONM for spinal surgeries below the C8-T1 level, upper extremity muscles are often used as control muscles, assuming homogeneous effects of deep anesthesia across muscle groups, supported by the findings of Deguchi et al^17^ who found no difference in the effect of increasing propofol target concentrations on upper and lower extremity amplitudes. Consequently, if amplitudes in the leg muscles decline, but amplitudes in HAND are unchanged, this is currently often considered to indicate impending neurological injury. The use of HAND Tc-mMEPs as references during surgery below C8/T1 could therefore increase the false-positive rate for leg muscle Tc-mMEPs when anesthetic depth increases.

Notably, the study by Deguchi et al^17^ differed from ours in the number of Tc-mMEP measurements per subject, the analytical approach, and the focus on target propofol concentration instead of pEEG.^17^ In contrast to the findings of Deguchi et al,^17^ our results show that deep anesthesia has less effect on upper extremity amplitudes than lower extremity amplitudes. This aligns with transcranial magnetic stimulation research showing differences in excitability between upper and lower limb muscles,^37^ and studies focusing on inhalational anesthetics indicating that Tc-mMEPs signals from the lower extremity muscles appear more sensitive to anesthetic induced depression than those of upper extremity muscles.^10,38^

Using HANDs as a reference with the amplitude reduction method can lead to inaccurate warnings, obscuring whether a decrease in lower extremity Tc-mMEP amplitude is due to spinal cord compromise or deep anesthesia. If leg amplitudes decrease but hand amplitudes do not, reassessing thresholds is advisable. Since we found no clinically relevant changes in Tc-mMEP thresholds during deeper anesthesia, this approach could reduce the frequency of false-positive warnings and improve monitoring reliability. Conversely, amplitude warnings from HANDs are unlikely to be caused by excessive anesthetic depth.

**Figure 2.**
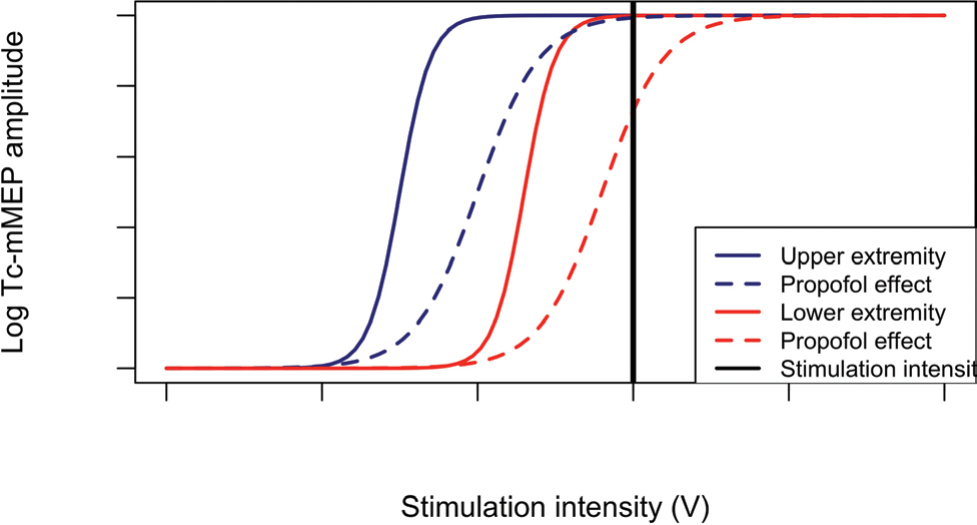
Propofol-mediated rotation of the stimulation intensity Tc-mMEP curve. Tc-mMEP indicates transcranial electrical stimulation muscle motor evoked potential.

A possible explanation for the differential effect on amplitudes is that the threshold intensity needed to elicit a Tc-mMEP is generally lower for upper extremity muscles than for lower extremity muscles. This is likely because the cortical area controlling hand movement is more superficial than that for leg muscles.^39^ Consequently, the stimulation intensity needed to achieve supramaximal Tc-mMEP amplitudes is lower for upper extremity muscles. If propofol causes a shallower slope of the stimulation-intensity curves, the impact on HAND amplitudes will be less pronounced than on leg muscles at the same stimulation intensity (Figure 2).

A second possible explanation is the greater number of direct connections between the motor cortex and the motor neurons innervating the hand compared to the leg.^40^ Scheufler and Zentner^41^ noted in their transcranial magnetic stimulation study that indirect waves may be suppressed by propofol-induced GABAergic inhibition within intracortical circuits. Since leg muscle innervation involves more interneuronal connections, this pathway may be more susceptible to changes in anesthetic depth induced by propofol. Further research is needed to elucidate the specific mechanisms underlying these differential effects on leg and HANDs.

This study has several limitations. First, data were collected exclusively from patients undergoing spinal surgery, which may limit the generalizability to other surgical contexts. Second, the study population was relatively young, potentially reducing applicability to older patients, as previous research has shown that Tc-mMEP thresholds tend to be higher in younger individuals.^42^ Finally, the high rate of missing data for our secondary objectives necessitated multiple imputation. However, results based on complete case data are included in the supplementary material for transparency.

**Table 5.** Effects of BIS Change on MEP Amplitude and AUC (Complex Model Including MAP and Propofol Concentration)

Muscle	Percentage change per 10 BIS points	95% CI	*P* value
Amplitude^a,b^			
AH	9.74	5.28–14.21	**<.001**
TA	7.28	2.82–11.76	**<.001**
GAS	12.08	7.61–16.56	**<.001**
HAND	2.87	−1.52 to 7.29	.200
AUC^a,b^			
AH	10.30	6.19–14.43	**<.001**
TA	7.44	3.31–11.58	**<.001**
GAS	11.94	7.81–16.10	**<.001**
HAND	3.22	−0.82 to 7.29	.119

Bold values indicate statistically significant values.

Abbreviations: AH, abductor hallucis muscle; AUC, area under the curve; BIS, bispectral index; CI, confidence interval; GAS, gastrocnemius muscle; HAND, hand muscle; TA, tibialis anterior muscle.

a Proportion of BIS = 40 transcranial electrical stimulation muscle motor evoked potential outcomes, corrected for side, muscle, and randomization group.

b Log transformed outcome data were added to the model, interaction between BIS and muscle was added. Models with mean arterial pressure and calculated plasma concentrations of propofol as additional variables.

This study quantified the relationship between depth of anesthesia and Tc-mMEPs, demonstrating a positive association between BIS and Tc-mMEP amplitude and AUC in leg muscles. Therefore, maintaining a relatively constant anesthetic depth should reduce the incidence of false-positive Tc-mMEP amplitude warnings. Future research should focus on the precise effects of anesthetic depth on D-and I-waves across various spinal levels, to deepen our understanding of the physiological mechanisms underlying the differential effects of anesthesia on the upper and lower extremity Tc-mMEP responses.

## DISCLOSURES

**Conflicts of Interest:** A. R. Absalom reports receipt of unrestricted research funding and/or reimbursement for consultancy from Philips (Eindhoven, the Netherlands) (all payments to institution), and is a trustee of the *British Journal of Anaesthesia* company. No other authors declared Conflicts of Interest. **Funding**: None. **This manuscript was handled by:** Markus W. Hollmann, MD, PhD.

## Supplementary Material


