# Supplementary material for: The Effects of Depth of Anesthesia on Muscle-Recorded Motor Evoked Potentials: A Prospective Observational Study
Source: Anesth Analg. 2025 Nov 20;142(4):741–50. doi: 10.1213/ANE.0000000000007777 (PMC12959600; doi:10.1213/ANE.0000000000007777)
Supplement: Supplementary file 1 [file ane-142-741-s001.pdf]

SUPPLEMENT A

Post-hoc power calculation

As our study was exploratory in nature, and there was no information available on parameters necessary to calculate sample size, we have not performed such a calculation in advance. A post-hoc calculation using the makeLmer and powersim functions from the simr package in R *(Green & MacLeod, 2016)* yielded 100% post-hoc power (95% CI: 83–100%) with the following specifications: 25 subjects, 100 observations per subject, a fixed intercept of -0.114 and the estimate of single main effect of continuous log BIS of 0.009, a random intercept variance of 0.116, and a residual standard variation of 0.498. These parameters were taken from the model output with Tc-mMEP amplitude that is reported in Table 2, rounded to the nearest third digit and were included in 20 simulations. Incrementally manipulating the estimate of continuous log BIS showed that power remained 100% until it was decreased to 0.001, at which point power was still estimated to be at 70% (95% CI: 45%–88%), indicating considerable robustness.
