# Supplementary material for: The Effects of Depth of Anesthesia on Muscle-Recorded Motor Evoked Potentials: A Prospective Observational Study
Source: Anesth Analg. 2025 Nov 20;142(4):741–50. doi: 10.1213/ANE.0000000000007777 (PMC12959600; doi:10.1213/ANE.0000000000007777)
Supplement: Supplementary file 2 [file ane-142-741-s002.pdf]

SUPPLEMENT B

Case example of amplitude decline when BIS decreased from 50 to 30.

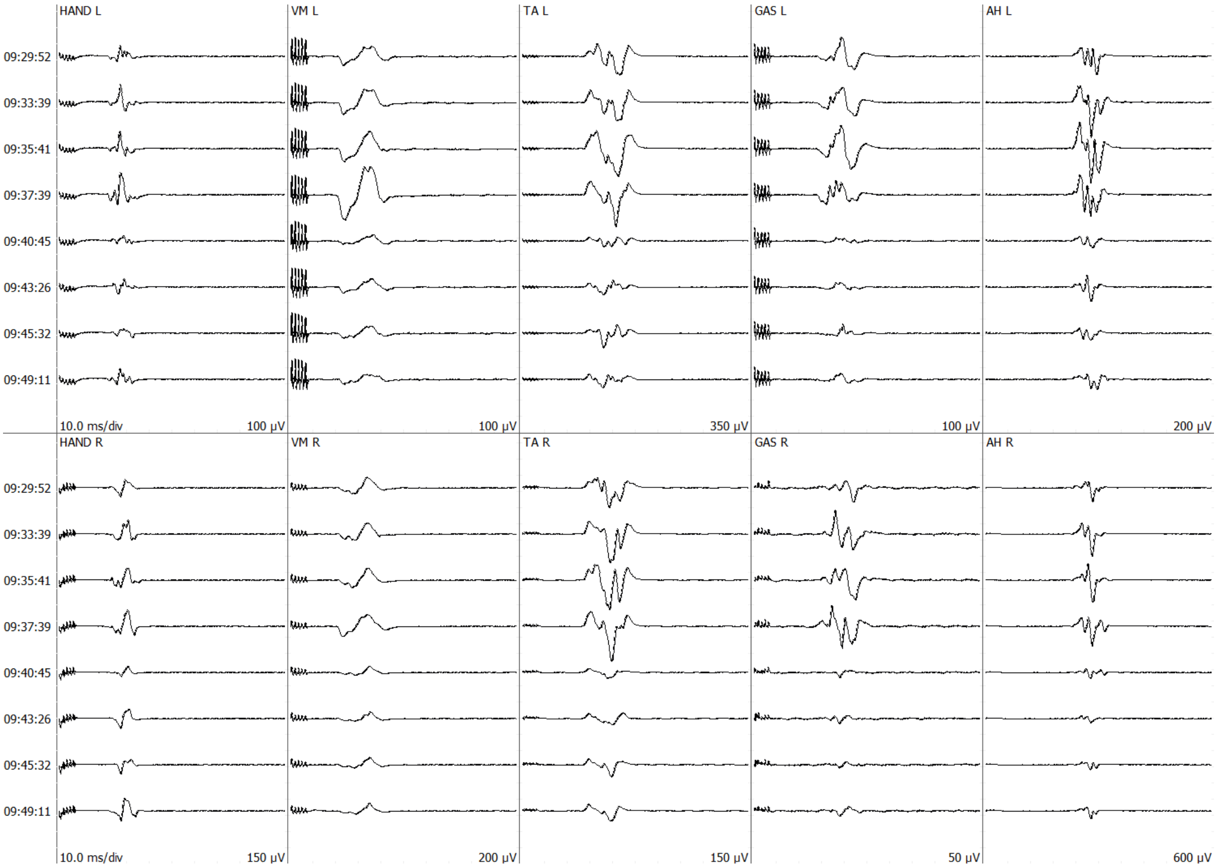

Abbreviations: VM= vastus medialis; TA= tibialis anterior; GAS= gastrocnemius; AH= abductor hallucis; L= left; R= right.
