# Supplementary material for: The Effects of Depth of Anesthesia on Muscle-Recorded Motor Evoked Potentials: A Prospective Observational Study
Source: Anesth Analg. 2025 Nov 20;142(4):741–50. doi: 10.1213/ANE.0000000000007777 (PMC12959600; doi:10.1213/ANE.0000000000007777)
Supplement: Supplementary file 3 [file ane-142-741-s003.pdf]

SUPPLEMENT C

Secondary objectives complete case analysis

Part I

Table 1. Complete cases model without plasma samples

Variable	AIC	Delta AIC	Conditional R^2
BIS	3080.125	9.622	0.380
Propofol target concentration	3070.503	0.00	0.371
Propofol calculated effect site concentration	3072.620	2.117	0.372
Propofol calculated plasma concentration	3089.554	19.051	0.369

A total of 1967 datapoints (=78% of the original dataset) per model were used for the analysis

Table 2 Complete case model with plasma samples

Variable	AIC	Delta AIC	Conditional R^2
BIS	670.025	11.128	0.394
Propofol target concentration	663.595	4.698	0.388
Propofol calculated effect site concentration	666.768	7.871	0.378
Propofol calculated plasma concentration	658.897	0.00	0.402
Propofol measured plasma concentration	675.008	16.111	0.376

A total of 395 datapoints (=16% of the original dataset) per model were used for the analysis

Part II

Table 3. Complete case analysis part B

		**Percentage change per 10 BIS points**	**95% CI**	**p-value**
**Amplitude*[1]**	AH	8.39	3.17 – 13.64	**0.002**
	TA	6.36	1.13 – 11.62	**0.017**
	GAS	11.20	6.74 – 17.28	**<0.001**
	HAND	0.59	-4.54 – 5.75	0.823
**AUC*[1]**	AH	9.28	4.47 – 14.12	**<0.001**
	TA	6.75	1.93 – 11.59	**0.006**
	GAS	11.26	6.42 – 16.13	**<0.001**
	HAND	1.27	-3.46 – 6.02	0.600

*Abbreviations; AUC, area under the curve; V, voltage; AH, abductor hallucis muscle; TA, tibialis anterior muscle; GAS, gastrocnemius muscle. *Proportion of BIS= 40 Tc-mMEP outcomes, corrected for side, muscle, and randomization group. [1]Log transformed outcome data was added to the model, interaction between BIS and muscle was added. Model with mean arterial pressure and calculated plasma concentrations of propofol as additional variables.*

A total of 1999 datapoints (=80% of the original dataset) were used for the analysis.
